# DeepSSETracer 2.0: Improved Deep Learning Model Performance for Protein Secondary Structure Segmentation from Cryo-EM Maps

**DOI:** 10.1145/3768322.3769026

**Published:** 2025-12-05

**Authors:** Bryan Hawickhorst, Thu Nguyen, Willy Wriggers, Jiangwen Sun, Jing He

**Affiliations:** 1 Department of Computer Science, Old Dominion University, Norfolk, Virginia, USA; 2 Department of Mechanical and Aerospace Engineering, Old Dominion University, Norfolk, Virginia, USA

**Keywords:** Deep learning, Cryo-EM, Protein, Secondary structure

## Abstract

DeepSSETracer is a method for segmenting protein secondary structure from medium-resolution (5–10Å) cryogenic electron microscopy (cryo-EM) density maps. We conducted experiments and ablation studies to examine the effects of normalization methods, max-pooling, activation functions, and loss calculation region on DeepSSETracer. By combining multiple technical improvements, the performance of the new version, DeepSSETracer 2.0, was significantly enhanced compared to DeepSSETracer 1.1. On a set of 77 test cases, the weighted average per-voxel F_1_ score increased from 62.1% to 70.3% for helix detection, and from 47.8% to 62.5% for β-sheet detection. While each of the five modifications in the network enhanced the detection of both helices and β-sheets, the improvement on β-sheets was even more pronounced. The ablation studies show that the most enhanced accuracy comes from the replacement of batch normalization with instance normalization, which accounts for increased F_1_ scores by 3% (helix) and 6.3% (β-sheet). These results show that relatively modest network tuning can significantly improve segmentation, suggesting that further incremental gains remain possible within the U-Net deep learning architecture.

## 1 INTRODUCTION

Cryo-electron microscopy (cryo-EM) with single-particle reconstruction has enabled routine high-resolution structure determination for many protein complexes. However, structural interpretation remains challenging when density maps are only available at medium resolution (5–10 Å) [1]. Cryo-electron tomography (cryo-ET) [2], combined with subtomogram averaging [3], has recently emerged as an alternative method for producing maps in this resolution range. Together, these developments highlight the need for reliable secondary structure prediction to aid structural interpretation of such maps.

At medium resolution, α-helices and β-sheets are discernible but often confounded by neighboring densities, making automatic secondary structure detection difficult. Deep learning was first applied to identify α-helices and β-sheets from medium-resolution cryo-EM maps in Li et al [4]. We later replaced the complicated architecture used in that work with a simpler U-Net architecture [5, 6], and we used an alternative loss function in DeepSSETracer 1.1 to improve detection performance [7]. While promising, DeepSSETracer 1.1 still had accuracy limitations, partially due to insufficient optimization of its network components.

We conducted experiments to study the effect of network components, including normalization methods, the number of max-pooling, activation functions, and the region size of the loss function. Previously, we trained the deep learning model using 1,246 map/structure pairs at 5–10 Å resolution, downloaded from the Electron Microscopy Data Bank (EMDB), along with their corresponding Protein Data Bank (PDB) structures [7]. We have now expanded the dataset to 1,849 nonredundant map/structure pairs by including all new entries (at the same resolution range) deposited between July 2018 and February 2025. With this expanded dataset and updated network components, we refined DeepSSETracer’s deep learning model, achieving better generalization and higher accuracy for secondary structure segmentation in cryo-EM density maps.

## 2 METHODS

To improve our deep learning model, we studied the effect of multiple changes in the network and expanded the dataset. Below, we outline these modifications and describe our training and evaluation process.

### 2.1 DeepSSETracer 1.1’s Deep Learning Model

DeepSSETracer 1.1 employed a 3D U-Net architecture [6–8]. Specifically, it used two max-pooling layers instead of the default three layers as used in the original 3D U-Net architecture [8]. To accommodate the network’s receptive field, loss was calculated after trimming 34 voxels (34 Å) from each dimension. The final binary segmentation mask and F_1_ score calculations used the same 34-voxel trim.

The network takes as input a box-cropped region of a 3D cryo-EM map and classifies each voxel as helix, β-sheet, or background. Two segmented 3D maps are produced, one for detected helix regions and the other for detected β-sheet regions.

### 2.2 DeepSSETracer 2.0’s Updated Architecture

DeepSSETracer 2.0 introduced an updated in-network normalization method, an additional max-pooling layer, a new activation function, and loss calculation over a larger output volume than in the 1.1 version. Below, we describe the motivation for each modification.

#### 2.2.1 In-network normalization.

DeepSSETracer 1.1 used batch normalization (BN) between each convolutional and activation layer [9]. In order to maximumly preserve molecular structural features, the network input is a box-cropped 3D volume, which is generally large. Limited by the 80 GB memory capacity of the NVIDIA A100 GPU, a batch size of 1 was used. However, BN performs poorly with low batch sizes [10]. We tested three alternatives in this study: instance normalization (IN), layer normalization (LN), and group normalization (GN). IN, which normalizes across spatial dimensions for each channel, has been widely adopted in computer vision, including nnU-Net [11, 12]. LN normalizes across both spatial and channel dimensions, while GN divides channels into groups and normalizes across spatial and group dimensions [10, 13]. IN provided the highest performance and stability (details in Section 3), and we adopted it as the new baseline before testing further changes.

#### 2.2.2 Max pooling layer.

β-sheets exhibit complex and highly varied conformations that are harder to resolve than helices. Gradient-weighted Class Activation Mapping (Grad-CAM) analyses showed that helices were detected early in the network, whereas β-sheets typically required deeper layers [14, 15]. These observations motivated us to add another max pooling layer and subsequent convolutional block, aiming to improve the abstraction and representation of sheet features.

#### 2.2.3 Activation function.

Changing activation functions requires minimal network modification. There is evidence that certain activation functions may improve convergence on vision tasks. We tested six activation functions. The Rectified Linear Unit (ReLU) activation function, the default in 3D U-Net, applies f(x) = max(0, x) and is widely used in computer vision. LeakyReLU introduces a small negative slope to ReLU below zero to avoid inactive neurons and has been adopted successfully in nnU-Net. Parametric ReLU (PReLU) generalizes ReLU by learning the negative slope parameter, and it has exhibited strong results across computer vision tasks [16]. The Gaussian Error Linear Unit (GELU) activation function provides a smooth gating of inputs, blending linear and nonlinear behavior, and has been widely adopted in modern vision and language models [17]. Mish uses a smooth non-monotonic activation that improves gradient flow and has shown improvements in image classification [18]. Swish (also known as SiLU) is a smooth, self-gated activation that has demonstrated improved convergence in convolutional networks [19]. Of these functions, PReLU performed the best on our dataset and was adopted in the updated model (details in Section 3).

#### 2.2.4 Loss region.

Given the receptive field size of DeepSSETracer 1.1, loss was calculated after cropping 34 voxels (34 Å) from each dimension of the output to eliminate potential artifacts from near-edge voxels. To provide a stronger training signal, we tested smaller trims of 16, 6, and 0 voxels on each dimension.

### 2.3 Dataset Preparation

DeepSSETracer 1.1 used 1,355 cropped 3D volumes from cryo-EM density maps with resolutions between 5 and 10 Å [7]. The cryo-EM density maps were downloaded from the EMDB along with their corresponding PDB structures. Of these, 1,246 entries were allocated for training, 62 for testing, and 47 for validation.

We expanded the dataset by downloading all maps deposited between July 2018 and February 2025 in the same resolution range and processed them following the procedure described by Mu et al. (2024) to generate box-cropped map/structure pairs. To reduce redundancy, we used MMseq2 to compute sequence identities for center-chain sequences and excluded entries with >70% identity to any chain in the original dataset [20].

We then calculated Q-scores, which measure atom resolvability, using the Q-Score plugin in ChimeraX [21, 22]. From these, we randomly selected 633 box-cropped map/structure pairs with average Q-scores above 0.25, yielding a final dataset of 1,849 training, 77 testing, and 57 validation entries.

### 2.4 Model Training

Models were trained in PyTorch using the Adam optimizer with a weight decay of 1e-3 [23]. Automatic mixed precision was enabled to reduce memory usage and training time. Due to the large size of some of the volumes in our dataset, we used a batch size of 1. We trained each model for 40 epochs, and the best checkpoint was selected based on the maximum mean helix and β-sheet F_1_ score on the validation set. All models were trained on a single NVIDIA A100 80 GB GPU. We tested learning rates of 1e-3, 1e-4, and 1e-5; 1e-4 consistently performed best and was used in all experiments.

We used a combined focal and dice loss function, previously shown to improve β-sheet segmentation in medium-resolution 3D cryo-EM maps [7]. We tested four values of γ in this loss function: γ = 0, 1, 2, and 5. We found this hyperparameter to be minimally impactful, so we used γ = 1 in all experiments.

## 3 RESULTS

We first present exploratory experiments testing alternative normalization methods, the number of max-pooling layers, activation functions, and loss calculation regions to guide the design of version 2.0. These experiments used the original DeepSSETracer 1.1 U-Net architecture as a baseline. The dataset comprised 1246 training cases and 47 validation cases. Based on results from the experiments, we then finalize the DeepSSETracer 2.0 architecture and compare it with DeepSSETracer 1.1 on the expanded 77-case test set, showing improved model performance. Finally, we perform an ablation study by reverting individual modifications in version 2.0 to assess their contributions. F_1_ scores in Tables 1 through 4 and Table 6 were reported as the mean ± standard deviation across five training runs using different seeds ranging from 5 to 9.

### 3.1 Exploratory Analysis of DeepSSETracer 1.1 Network Modifications

We explored alternative normalization methods, activation functions, network depths, and trim sizes, starting from the original network in DeepSSETracer 1.1. Each experiment altered one component while keeping all others constant. Because BN showed training instability with a batch size of 1 (as detailed in 3.1.1), we adopted the highest-performing normalization, IN, as the new baseline for all subsequent comparisons in 3.1.

All analysis results in this section are reported on the 47-case validation set that was used in the original training of DeepSSETracer 1.1. Although this dataset differs from the 77-case set used in Sections 3.2 and 3.3, the experiments based on the same validation set clearly showed performance differences for different network component changes. To evaluate the performance of each experiment, we first calculated per-voxel F_1_ score over the center region (with 34-voxel trim in each dimension) for each case in the validation set. We then obtained a weighted average F_1_ score over all 47 cases in the validation set using the percentage of helix or β-sheet voxels in each case as the weight. In this section, the weighted F_1_ score across the validation set was reported as an indication of the performance in each experiment.

#### 3.1.1 In-network normalization.

DeepSSETracer 1.1 utilized batch normalization. We sampled alternative normalization functions LN, IN, and GN. GN was tested with various group sizes ranging from 2 to 32. Since different group sizes showed similar performance, the result was reported for G = 4. Overall, IN achieved the highest performance; compared to BN, IN improved helix F_1_ score by 3.9% and β-sheet F_1_ by 5.7% (Table 1). All alternative normalization methods outperformed BN, and their standard deviations are lower than that of BN. Our experiments of the four normalization methods show the weakness of BN at a low batch size of 1. IN appears to be the best overall among the four, in terms of the accuracy and stability for the batch size of 1.

#### 3.1.2 Max pooling layer.

We tested the impact of adding a third max pooling layer to DeepSSETracer 1.1. Since IN was shown as the best performing normalization method on our dataset (Table 1), it was adopted in this max-pooling layer experiment. Compared to the two-layer baseline, this modification improved helix F_1_ by 1.3% and β-sheet F_1_ by 1.1% (Table 2). Although modest, the improvement was consistent across both classes.

#### 3.1.3 Activation function.

We compared the use of six activation functions in the network of DeepSSETracer 1.1 (Table 3). In DeepSSETacer 1.1, ReLU was used as the activation function. In this experiment, IN was used as the normalization method, since it showed an advantage over BN (see 3.3.1). Although most alternatives achieved similar performance, with only small variations in helix segmentation, PReLU provided the most consistent gains, improving β-sheet F_1_ by 1.4% (Table 3). Although modest, this improvement was computationally inexpensive and we adopt PReLU for the new version of DeepSSETracer.

#### 3.1.4 Loss calculation region.

Finally, we tested the effect of trimming the region used for loss calculation. Due to the network’s receptive field, edge voxels lack full context and are therefore less accurately classified. However, ignoring edge voxels when calculating loss reduces the amount of labeled data. This raises the question of whether it is beneficial to include the loss of those voxels near the edges during training. We tested trims of 34, 16, 4, and 0 voxels. Compared to the default 34-voxel trim, in which 17 voxels were removed from each end of a dimension. 0-voxel trim for loss calculation improved helix F_1_ by 1.6% and β-sheet F_1_ by 4.2%, increasing the mean F_1_ from 56.4% to 59.4% (Table 4). Note that the per-voxel F_1_ score in Section 3.1 measures the volume after the 34-voxel trim, without those voxels near edges. Our experiments show that reducing or eliminating trimming in loss calculation consistently improves performance for voxels in the central region (after the 34-voxel trim), especially for β-sheet detection. This makes sense, since some β-sheets are large and often have various shapes, more complicated than helices. This result suggests that including all voxels, including those voxels near the edges that are potentially less accurately classified, in loss calculation, still helps the classification of those in the center region.

### 3.2 DeepSSETracer 2.0

DeepSSETracer 2.0 model was trained using a modified network based on our analysis in Section 3.1 regarding normalization, max pooling, activation, and loss calculation. The model was produced using IN, PreLU, an additional max-pooling layer, 0-voxel trim in loss calculation, and a larger dataset than what was used in producing the DeepSSETracer 1.1 model. DeepSSETracer 2.0 model was trained using the 1849-case dataset and selected with the 57-case validation set. To compare with the DeepSSETracer 1.1 model that was trained using the original network and 1246-case training set, both versions were tested using the 77-case set.

With its updated architecture, DeepSSETracer 2.0 showed significant improvement in both helix and β-sheet detection. If both models of the two versions are evaluated in the center region with the 34-voxel trim, as for DeepSSETracer 1.1, the per-voxel F_1_ score across all 77 test cases improved from 62.1% to 71.6% for helix and from 47.8% to 63.9% for β-sheet detection (Table 5). Note that the results in Table 5 used the 77-case test set, not the 47-case validation set that was only used for analysis purposes (Table 1–4). The significant jump in accuracy shows the effectiveness of the four modifications in the network. In addition to the improved accuracy at the center region of the map, DeepSSETracer 2.0 produces larger segmentations, as it only trims off 10 voxels in each dimension before the final output. If Version 2.0 is measured on the larger segment with the 10-voxel trim, the per-voxel F_1_ scores increased by 8.2% for helices and 14.7% for β-sheets (rows 1 and 3 in Table 5). The mean F_1_ scores across helices and β-sheets increased from 55.0% to 66.4%. Notably, β-sheet detection benefited most from the updated network elements. DeepSSETracer 2.0 also demonstrates significantly less degradation in performance near volume edges compared to DeepSSETracer 1.1, since the per-voxel F_1_ score only reduced slightly between 0.3% to 0.6% when the trim is reduced from 34 to 10.

**Figure 1:**
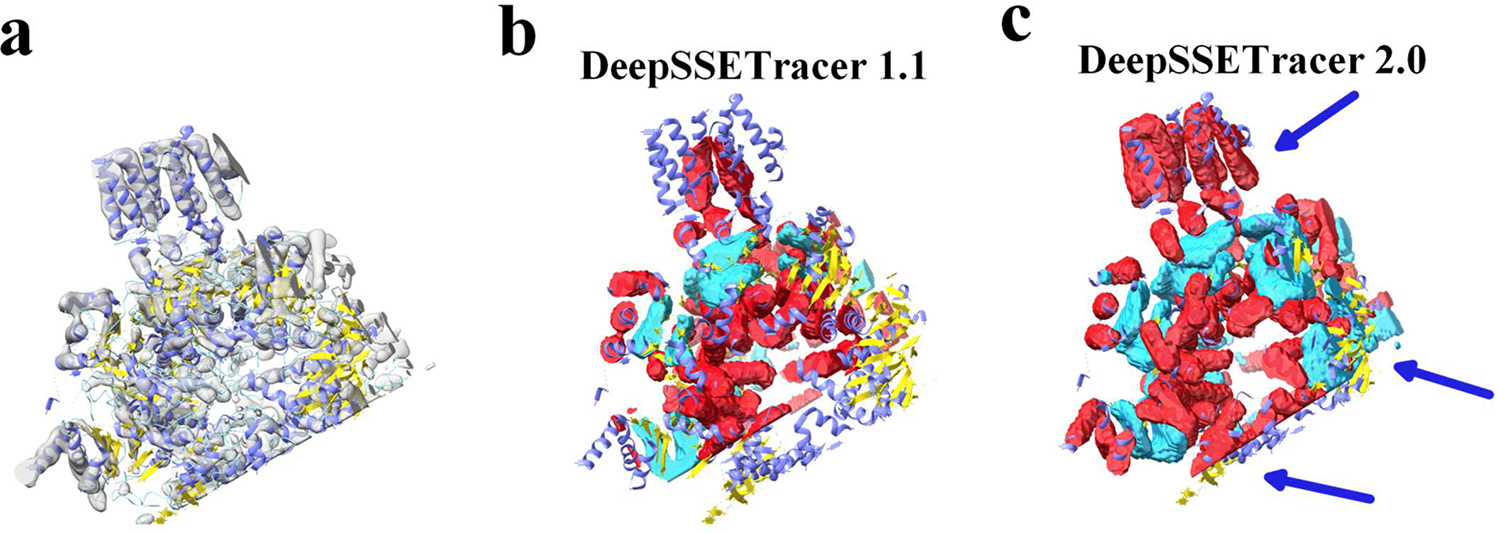
Segmentation of secondary structure elements, in a box-cropped region (size 120×120×112) around Chain 3 of molecular entry with PDB ID 4CR2. (a) The box-cropped map from the cryo-EM map with EMDB ID EMD-2594 (7.7 Å resolution, shown in transparent gray) and its corresponding structure shown in ribbon (helix: lavender, sheet: yellow) (b) Segmentation result using DeepSSETracer 1.1 (c) Segmentation result using DeepSSETracer 2.0. The detected helix (red) and β-sheet (cyan) regions are shown in (b) and (c). Arrows point to the secondary structure regions that were missed using DeepSSETracer 1.1.

In an example around Chain 3 of the PDB structure (PDB ID 4CR2) and its corresponding cryo-EM map with ID EMD-2594, a case in the expanded test set, DeepSSETracer 2.0 corrected some mispredictions resulting from version 1.1 (Figure 1). It also identifies larger regions of helix and β-sheet (blue arrows), demonstrating more robust segmentation compared to version 1.1.

### 3.3 DeepSSETracer 2.0 Ablation Study

We conducted an ablation study using the DeepSSETracer 2.0 architecture, hyperparameter settings, and the new training dataset containing 1849 data, then reverted one modification at a time back to its 1.1 setting. For each of the five experiments, five models were trained using five seeds. The per-voxel F_1_ scores in the center region (F1 trim=34) were calculated across the 77 cases. The normalization function had the largest effect: Replacing instance normalization (used in Version 2.0) with batch normalization (in Version 1.1) reduced the mean F_1_ score from 71.9% to 68.9% for helices and from 63.9% to 57.6% for β-sheets (Table 6).

Although each reverted modification reduced detection accuracy, helix detection was less affected than β-sheet detection. The standard deviation among the five models was largest for β-sheet detection when using ReLU (0.8%) or the smaller training set (0.7%), indicating less stable performance.

## 4 CONCLUSION

In summary, we found that a relatively modest tuning of our network settings substantially improved secondary structure segmentation. Overall, the voxel-level F_1_ scores of DeepSSETracer 2.0 increased from 62.1% to 70.3% for helix detection and from 47.8% to 62.5% for β-sheet detection, based on a test of 77 cases. The enhancement in the network settings enhanced the detection of β-sheet more than for helix, suggesting that accurate detection of β-sheets requires fine design of the network and training.

The ablation studies of six modifications in developing the new version of DeepSSETracer from the baseline version show that each modification contributed to the improved model. However, the largest improvement came from replacing the normalization method. Instance normalization proved more effective than batch normalization which showed the weakness at a low batch size. Additionally, using a smaller trim size for the calculation of loss, adding a max-pooling layer, and adopting PReLU activation all contributed to enhanced model performance.

Most models at 5–10 Å resolution are derived from fitting known templates into lower-resolution maps. We did not differentiate between rigid-body and flexible fitting. Instead, we used the Q-score metric to bias our data toward good matches between maps and fitted models [22]. While it is important to exclude poorly matched regions from training, Q-scores are less reliable at medium resolution (5–10 Å) than at high resolution [24]. Future work should aim to identify more suitable metrics for selecting high-quality matches. One candidate is the cylindrical score [25, 26]; however, it currently applies only to helices, not β-sheets, and thus could not be used in this study. Developing an analogous metric for β-sheets would be valuable but challenging, as their densities are much weaker than those of helices at medium resolution (5–10 Å), making accurate prediction more difficult.

## Figures and Tables

**Table 1:** Validation set weighted-average F_1_ scores (%) of the modified DeepSSETracer 1.1 with alternative normalization methods

Normalization	Helix	β-sheet	Mean
BN	59.5 ± 1.9	43.7 ± 1.4	51.6 ± 0.7
LN	62 ± 0.3	49 ± 0.5	55.5 ± 0.3
GN (G=4)	62.2 ± 0.2	48.8 ± 0.8	55.5 ± 0.3
IN	63.4 ± 0.4	49.4 ± 0.8	56.4 ± 0.2

**Table 2:** Validation set weighted-average F_1_ scores (%) of the modified DeepSSETracer 1.1 with IN and alternative max pooling layers

# Max Pools	Helix	β-sheet	Mean
2	63.4 ± 0.4	49.4 ± 0.8	56.4 ± 0.2
3	64.7 ± 0.7	50.5 ± 0.8	57.7 ± 0.4

**Table 3:** Validation set weighted-average F_1_ scores (%) of the modified DeepSSETracer 1.1 with IN and alternative activation functions

Activation	Helix	β-sheet	Mean
ReLU	63.4 ± 0.4	49.4 ± 0.8	56.4 ± 0.2
GELU	63.4 ± 0.3	49.6 ± 0.6	56.5 ± 0.4
LeakyReLU	63.4 ± 0.7	49.3 ± 0.3	56.4 ± 0.4
Mish	62.7 ± 0.3	48.7 ± 0.5	55.7 ± 0.3
SiLU	63.1 ± 0.4	48.8 ± 0.7	55.9 ± 0.3
PReLU	63.6 ± 0.3	50.8 ± 0.7	57.2 ± 0.4

**Table 4:** Validation set weighted-average F_1_ scores (%) of the modified DeepSSETracer 1.1 with IN and alternative trim sizes for loss calculation.

Loss Region	Helix	β-sheet	Mean
Trim = 34	63.4 ± 0.4	49.4 ± 0.8	56.4 ± 0.2
Trim =16	65.0 ± 0.3	52.9 ± 0.4	59.0 ± 0.3
Trim = 6	65.3 ± 0.4	53.5 ± 0.9	59.4 ± 0.3
Trim = 0	65 ± 0.2	53.6 ± 0.3	59.4 ± 0.2

**Table 5:** Weighted F_1_ scores (%) of DeepSSETracer 1.1 and DeepSSETracer 2.0 on the 77-case test set

Model	Helix	β-sheet	Mean
v1.1 (F1 trim = 34)	62.1	47.8	55.0
v2.0 (F1 trim = 34)	71.6	63.9	67.7
v2.0 (F1 trim = 10)	70.3	62.5	66.4

**Table 6:** Ablation study weighted F_1_ scores (%) on the 77-case test set for DeepSSETracer 2.0

Reverted Change	Helix	β-sheet	Mean
None (v2.0)	71.9 ± 0.3	63.9 ± 0.5	67.9 ± 0.2
Loss Trim = 34	70.2 ± 0.6	61.2 ± 0.5	65.7 ± 0.5
# Max Pools = 2	70.3 ± 0.3	61.5 ± 0.5	65.9 ± 0.4
ReLU	70.8 ± 0.2	62 ± 0.8	66.4 ± 0.3
BN	68.9 ± 0.5	57.6 ± 0.6	63.2 ± 0.4
v1.1 Training Set (1246)	71.6 ± 0.2	61.6 ± 0.7	66.6 ± 0.4

## CCS CONCEPTS

• Machine Learning • Computational Biology
